# Progressive neurodegeneration, motor decline, and premature mortality in aging Ngly1 deficient rats

**DOI:** 10.1186/s13023-026-04261-1

**Published:** 2026-02-20

**Authors:** Lei Zhu, Selina Dwight, William F. Mueller, Becky Schweighardt

**Affiliations:** 1 Grace Science, LLC, Menlo Park, CA 94025 USA; 2 Grace Science Foundation, Menlo Park, CA 94025 USA

**Keywords:** NGLY1, NGLY1 Deficiency, Rare diseases, Motor function, CNS, Mortality

## Abstract

**Background:**

N-glycanase 1 (NGLY1) Deficiency is an ultra-rare autosomal recessive disorder of deglycosylation caused by loss-of-function mutations in the *NGLY1* gene. Patients present with developmental delay, intellectual disability, hyperkinetic movement disorder, elevated liver enzymes, (hypo)alacrima, and peripheral neuropathy. Despite supportive care, many experience early neurological deterioration, with loss of previously attained motor skills by adolescence. Additionally, life-threatening complications are not uncommon, and the published median lifespan of patients is ~13 years. The pathophysiology of NGLY1 Deficiency remains poorly understood, in part due to limited long-term studies in animal models. Notably, *Ngly1*⁻^/^⁻ mice (C57BL/6) are embryonically lethal, and prior characterization of *Ngly1*⁻^/^⁻ rats was restricted to young adult rat (~7 months old), leaving late-onset phenotypes and potential lifespan reduction unexplored.

**Methods:**

In the study reported here, longitudinal assessments of phenotypes in *Ngly1*⁻^/^⁻ rats were conducted alongside *Ngly1*⁺^/^⁻ and *Ngly1*⁺^/^⁺ control rats. Survival, motor function, biochemical biomarkers, and brain histopathology were examined in the rats from approximately 6 months to 17–18 months of age.

**Results:**

*Ngly1*⁻^/^⁻ rats exhibited markedly reduced lifespan, progressive neurological decline, and decreased quality of life compared with *Ngly1*⁺^/^⁻ and *Ngly1*⁺^/^⁺ rats. By 9–10 months of age, ~50% of the *Ngly1*⁻^/^⁻ rats had either died or met humane euthanasia criteria due to a severe decline in health. Surviving animals displayed phenotypes mirroring human NGLY1 Deficiency disease progression, such as worsening motor deficits (~92% reduction in rotarod latency and ~82% reduction in rearing) and wide-spread neuroinflammation in multiple brain regions. In contrast, *Ngly1*⁺^/^⁻ and *Ngly1*⁺^/^⁺ littermates remained healthy and exhibited normal lifespan and aging profiles. Furthermore, histopathological examination of *Ngly1*⁻^/^⁻ rats identified significant neuropathological abnormalities that were not present in the control cohorts, including loss of peripheral axons and spinal motor neurons.

**Conclusion:**

The findings reported here demonstrate that *Ngly1*⁻^/^⁻ rats recapitulate the severe, progressive course of NGLY1 Deficiency, including neurodegenerative deterioration, motor deficits, and premature mortality. This extended longitudinal assessment of *Ngly1*⁻^/^⁻ rats provides important insights into disease progression and the shortened lifespan reported for human patients.

**Supplementary Information:**

The online version contains supplementary material available at 10.1186/s13023-026-04261-1.

## Introduction

NGLY1 Deficiency (OMIM #615273) is an ultra-rare autosomal recessive genetic disease caused by biallelic mutations in the *NGLY1* gene. *NGLY1* encodes N-glycanase 1, a conserved cytosolic enzyme that removes N-linked glycans from misfolded glycoproteins during endoplasmic reticulum-associated degradation (ERAD) [1]. Loss of NGLY1 disrupts this critical protein quality-control pathway, leading to accumulation of undegraded glycoproteins and widespread cellular stress [2–5]. Clinically, NGLY1 Deficiency presents as a suite of complex neurodevelopmental phenotypes with global developmental delay and/or intellectual disability, a hyperkinetic movement disorder, transient elevation of liver transaminases, (hypo)alacrima (insufficient or absent tear production), and a chronic diffuse sensorimotor neuropathy that affects the nerves in a length-dependent manner. As affected children grow, a characteristic hyperkinetic movement disorder (chorea/dystonia) emerges frequently alongside epilepsy [6]. Peripheral neuropathy develops progressively, often manifesting as areflexia and distal weakness, and other musculoskeletal complications such as scoliosis and contractures can arise [7–9]. Notably, while early mortality was not prominent in initial case studies [10, 11], recent longitudinal data suggest a limited lifespan in NGLY1 Deficiency patients, with a median age of death of 13 years reported in 2022 [9] and a more current estimate of 14.6 years [unpublished data, Grace Science Foundation]. While the etiology of NGLY1 Deficiency is not fully understood, reports have described neuronal loss in patients [19, 24] and found evidence of aggregation in human neurons [2]. Together, these observations hint at mechanisms that may underlie the progressive neurodegeneration seen in NGLY1 Deficiency.

Animal and cellular models are critical for understanding disease mechanism and for testing therapies, yet modeling NGLY1 Deficiency has been challenging. *Ngly1*⁻^/^⁻ mice in a C57BL/6 background exhibit embryonic lethality [12], precluding postnatal studies prior to the development of hybrids and inducible models [12, 15, 24, 25]. Mouse models created in backgrounds different from the C57BL/6 background (e.g. JF1, ICR) reproduce aspects of some clinical phenotypes, but require additional breeding, crossing, or knock-out inducing injections. Neuronal studies of these mice identified ubiquitinated protein accumulation in the hippocampus and thalamus. These findings exhibited some similarity to those in human tissue or neuronal cell lines, where protein aggregates and increases in aggresomes indicated defects in protein degradation [2, 15, 19, 24].

Given the limitations in mice, an *Ngly1*⁻^/^⁻ rat model was developed. This model enabled postnatal and longitudinal investigations without additional injections or cross breeding [13]. The *Ngly1*⁻^/^⁻ rat model exhibited high early mortality (70%) within the first three weeks after birth that stabilized after weaning at 21 days. Surviving *Ngly1*⁻^/^⁻ rats recapitulated key clinical features of NGLY1 Deficiency, including failure to thrive, peripheral neuropathy, gross motor defeiciencies, hyperkinetic movements, EEG abnormalities, sleep disturbances, convulsions, and delayed cognitive development [13, 14, 26].

Previous histopathological analyses in young adult *Ngly1*⁻^/^⁻ rats (up to ~ 7 months old) revealed neurodegenerative changes such as neuron loss in the thalamus, astrogliosis, microgliosis, and peripheral nerve axonopathy [15]. While providing valuable insights into disease pathology, these early studies were not designed to follow adult animals. Consequently, little is known about the trajectory of NGLY1 Deficiency into late adulthood in an animal model, including whether new phenotypes emerge or if the pathology worsens with age. This gap in understanding is relevant given that some NGLY1 Deficiency patients live beyond their teenage years despite the shortened mean lifespan associated with the disease [9].

This manuscript reports on an aging study in the *Ngly1*⁻^/^⁻ rat model to inform late-stage disease mechanisms with the eventual aim of developing and evaluating interventions that slow disease progression and extend lifespan. A longitudinal study of *Ngly1*⁻^/^⁻ rats was conducted to help characterize the course of NGLY1 Deficiency in aging animals. In this study, a cohort of homozygous *Ngly1*⁻^/^⁻ rats was followed alongside *Ngly1*⁺^/^⁻ (heterozygous) and *Ngly1*⁺^/^⁺ (wild-type) littermate control cohorts beginning at ~ 6 months of age through 17–18 months of age. Throughout this period, animals underwent periodic evaluations of motor behavior (rotarod, open field locomotor activity / rearing, and functional observational battery), assessments of levels of the NGLY1 Deficiency biomarker, GlcNAc-Asn (GNA) in both plasma and CSF [16], and clinical indicators of health (body weight, survival, cage-side/clinical observation, hematology, and clinical chemistry). At end of life, comprehensive histopathological examinations were performed on each cohort, focusing on the nervous system (brain, spinal cord, peripheral nerves) and other organs relevant to NGLY1 Deficiency disease pathology. Here, we report that NGLY1 Deficiency in a rat model of the disease leads to severe premature aging phenotypes, including significant motor decline and late-onset neuromuscular deterioration, as well as mid-life mortality. The *Ngly1*⁻^/^⁻ rat therefore serves as an important model of the human disease with respect to both severity and progression. Our findings extend the phenotypic characterization of *Ngly1*⁻^/^⁻ animals beyond early adulthood and underscore the utility of this rat model for studying the pathogenesis and treatment of NGLY1 Deficiency in its advanced stages.

## Results

### *Ngly1*⁻^/^⁻ rats exhibit progressive neurological phenotypes, reduced body weight, and premature mortality as they age

**Fig. 1:**
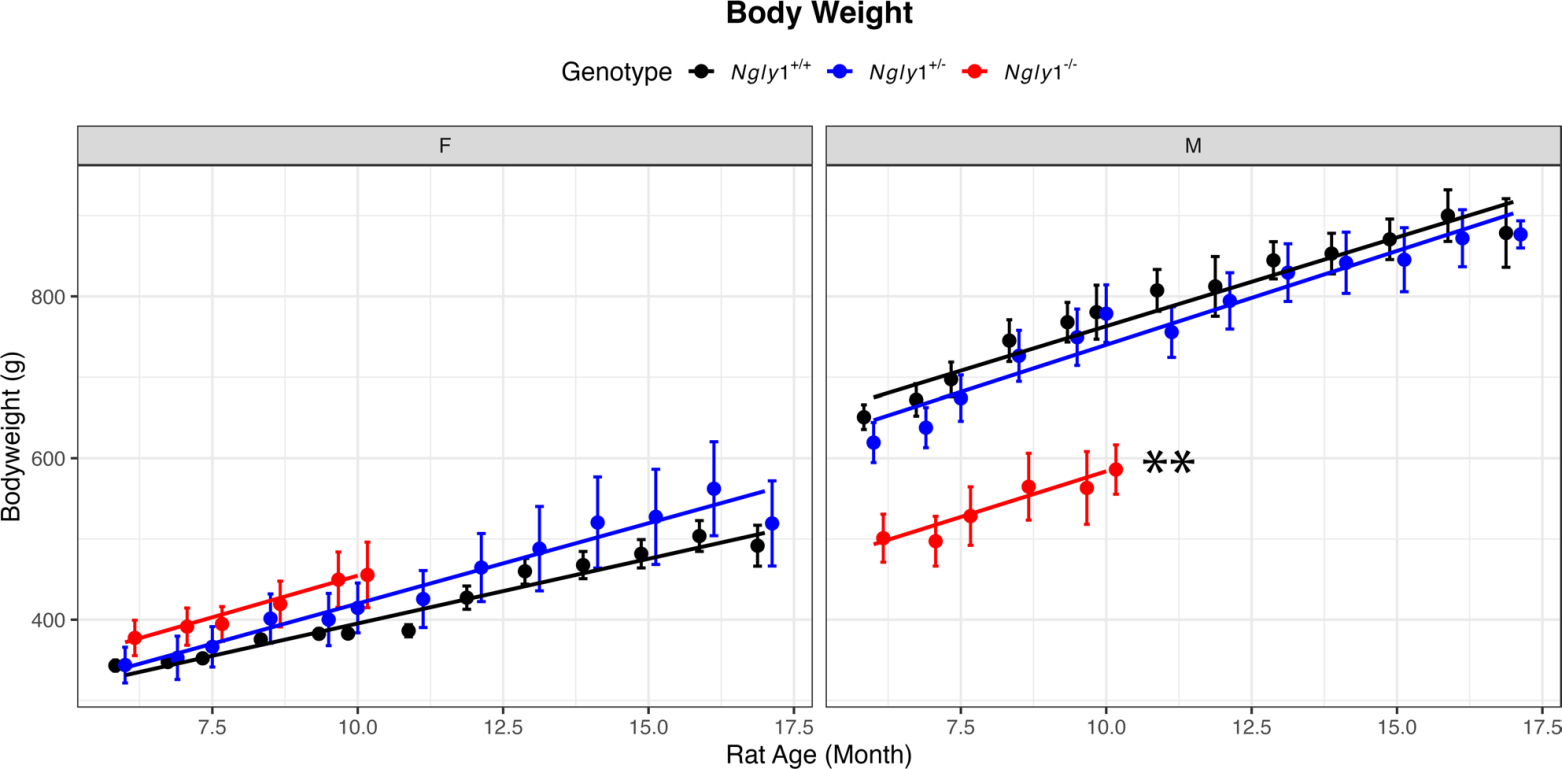
Body Weight Growth Curve of *Ngly1*⁺^/^⁺, *Ngly1*⁺^/^⁻, and *Ngly1*⁻^/^⁻ rats Over Study Course. Body weight was recorded monthly from 6 months of age until humane euthanasia or study termination for *Ngly1*⁺^/^⁺ (N = 10), *Ngly1*⁺^/^⁻ (N = 10), and *Ngly1*⁻^/^⁻ (N = 18) rats. Data are presented as mean ± SEM for each group at each recorded time point. **P < 0.01, Linear mixed model

At study initiation (~ 6 months of age), animals were assigned to one of three cohorts: (1) wild-type (*Ngly1*⁺^/^⁺; *N* = 10, 5 males/5 females), (2) heterozygous (*Ngly1*⁺^/^⁻; *N* = 10, 5 males/5 females), or (3) homozygous (*Ngly1*⁻^/^⁻; *N* = 18, 9 males/9 females). Additional animals were included in the *Ngly1*⁻^/^⁻ group to account for anticipated mortality-related loss of animal number. Male *Ngly1*⁻^/^⁻ rats weighed significantly less than *Ngly1*⁺^/^⁺ and *Ngly1*⁺^/^⁻ males throughout the study (*p* < 0.05, linear mixed model [LMM]), whereas female *Ngly1*⁻^/^⁻ body weights did not differ from their female controls (Fig. 1).

**Fig. 2:**
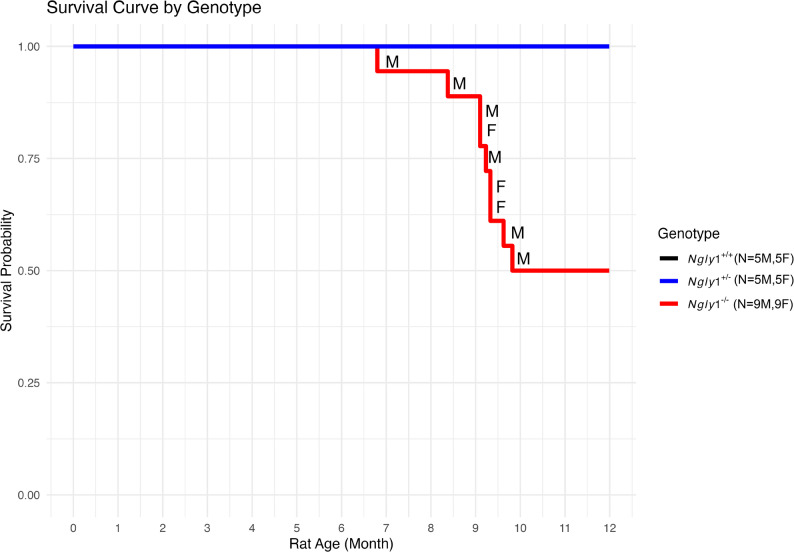
Survival Curve of *Ngly1*⁺^/^⁺, *Ngly1*⁺^/^⁻, and *Ngly1*⁻^/^⁻ rats. A Kaplan–Meier survival curve for the three rat study cohorts was plotted beginning at PND 21 (weaning) through12 months. The original number of rats for each genotype is indicated in the figure legend. The sex of each deceased rat was indicated as M (male) or F (female) to indicate when each individual animal died or was euthanized. All remaining *Ngly1*⁻^/^⁻ rats (along with 2 *Ngly1*⁺^/^⁺ and 2 *Ngly1*⁺^/^⁻ rats as controls) were humanely euthanized at 12 months of age due to deteriorating health conditions, and their tissues preserved. Note that *Ngly1*⁺^/^⁺ & *Ngly1*⁺^/^⁻ survival curves are overlapping so may not both be visible

Survival was assessed for each rat in the 3 cohorts (Fig. 2). Based on prior observations in *Ngly1*⁻^/^⁻ rats, litter sizes were not counted during the first few postnatal days to avoid disturbing the nests and potentially increasing postnatal mortality. As a result, overall survival in the postnatal period from time of birth to PND 15 could not be assessed. After genotyping at postnatal day (PND) 15 and weaning at PND 21, the survival was 100% in all genotypes until ~ 7 months of age, at which point *Ngly1*⁻^/^⁻ rats exhibited a premature mortality (natural or necessary euthanasia) that was not observed in either *Ngly1*⁺^/^⁺ or *Ngly1*⁺^/^⁻ cohorts. One of 18 *Ngly1*⁻^/^⁻ rats (~ 6%) died at PND 207 (~ 7 months). Between PND 255 and 299 (~ 8.5–10 months of age), four (22%) more *Ngly1*⁻^/^⁻ rats died and another 4 *Ngly1*⁻^/^⁻ rats (22%) required humane euthanasia due to severe decline in cage side/clinical observations. The animals found dead were observed to have uncoordinated gait, weakness, tooth grinding, and/or splayed limbs prior to their death, symptoms similar to previously described *Ngly1*⁻^/^⁻ rat phenotypes [13, 14, 26]. The cause of death was therefore reported as *Ngly1*⁻^/^⁻-related neurodegeneration [13, 14]. The remaining 10 *Ngly1*⁻^/^⁻ animals were humanely euthanized at 12 months as they showed various signs of severe health deterioration, and their tissues preserved.

### *Ngly1*⁻^/^⁻ Rats exhibit severe and persistent motor deficits in the rotarod assessment

**Fig. 3:**
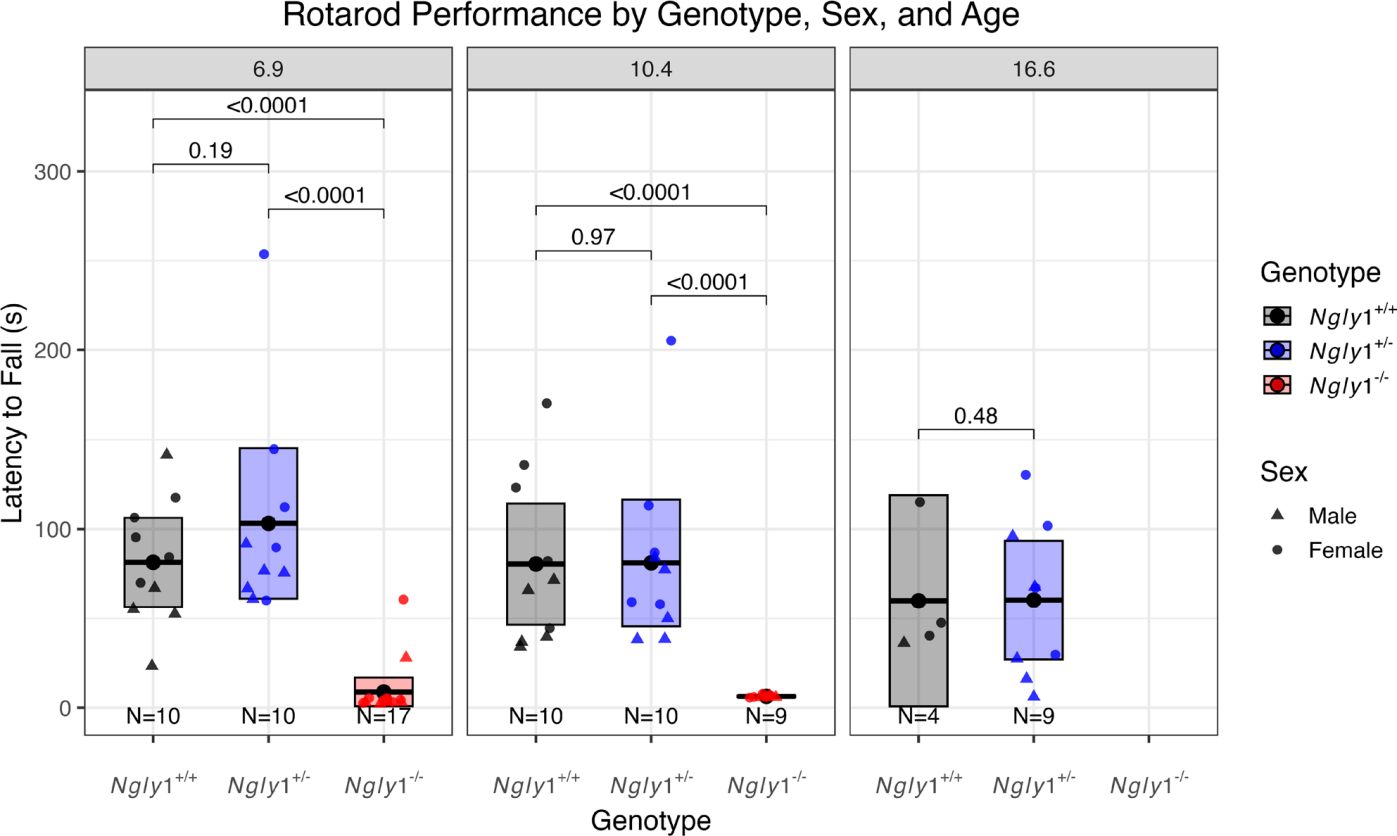
Rotarod Performance of *Ngly1*⁺^/^⁺, *Ngly1*⁺^/^⁻, and *Ngly1*⁻^/^⁻ rats Over Study Course. Rats were tested for latency to fall on rotating rotarod at 6.9, 10.4, and 16.6 months of age (all *Ngly1*⁻^/^⁻ rats were terminated by 12 months of age and were therefore only tested at the first two time points). A single conditioning rotarod session was performed prior to the actual testing. Each animal was evaluated for its ability to remain on the accelerating rotarod (latency to fall) in 3 trials with at least 15 min between trials. The average of all 3 trials is reported for each animal, and the median latency to fall per group is indicated as a dot-line within the box plot; the upper and lower edges represent 95% confidence interval (CI) (Mean ± CI). The p-value is labeled between genotypes at each time points and the number of animals (N) is indicated under each boxplot. Statistics: linear mixed model. M, male; F, female

The rotarod test measures a rodent’s latency to fall from a rotating rod and is a measure of motor function and has previously been used to characterize motor defects in the *Ngly1*⁻^/^⁻ rats [13, 14, 17, 18]. Rotarod performance was assessed at 6.9 and 10.4 months of age for *Ngly1*⁺^/^⁺, *Ngly1*⁺^/^⁻, and *Ngly1*⁻^/^⁻ rats and was determined to be significantly impaired in *Ngly1*⁻^/^⁻ rats compared to both *Ngly1*⁺^/^⁺ and *Ngly1*⁺^/^⁻ rats (Fig. 3). At ~ 6.9 months of age, *Ngly1*⁻^/^⁻ rats exhibited a latency to fall of 8.9 ± 3.7 (*N* = 17) seconds (s), which was significantly shorter than *Ngly1*⁺^/^⁺ (81.4 ± 11.0 s, *N* = 10, *p* < 0.0001, LMM) and *Ngly1*⁺^/^⁻ (103.2 ± 18.6 s, *N* = 10, *p* < 0.0001, LMM) rats. Similarly, at 10.4 months, *Ngly1*⁻^/^⁻ rats maintained significantly lower performance (6.4 ± 0.2 s, *N* = 9) than *Ngly1*⁺^/^⁺ and *Ngly1*⁺^/^⁻ rats, which exhibited sustained motor coordination over time with latencies of 80.4 ± 10.9s (*N* = 10) and 81.1 ± 11.6s (*N* = 10), respectively (*p* < 0.0001, LMM). No significant differences in rotarod performance were observed between *Ngly1*⁺^/^⁺ and *Ngly1*⁺^/^⁻ rats at any tested time point, including at 16.6 months of age, indicating that the severe motor deficit is specific due to loss of NGLY1. Data from *Ngly1*⁻^/^⁻ rats were not available for a comparison of rotarod performance at 16.6 months due to the premature mortality of some animals and the significant deterioration of the surviving animals. Interestingly, male *Ngly1*⁺^/^⁺ and *Ngly1*⁺^/^⁻ rats exhibited shorter rotarod latency to fall compared to their female counterparts, potentially reflecting the impact of greater body weight on motor performance [27].

### *Ngly1*⁻^/^⁻ Rats Show Significantly Reduced Locomotor Activity and Rearing at Compared with *Ngly1*⁺^/^⁺ and *Ngly1*⁺^/^⁻ Rats

**Fig. 4:**
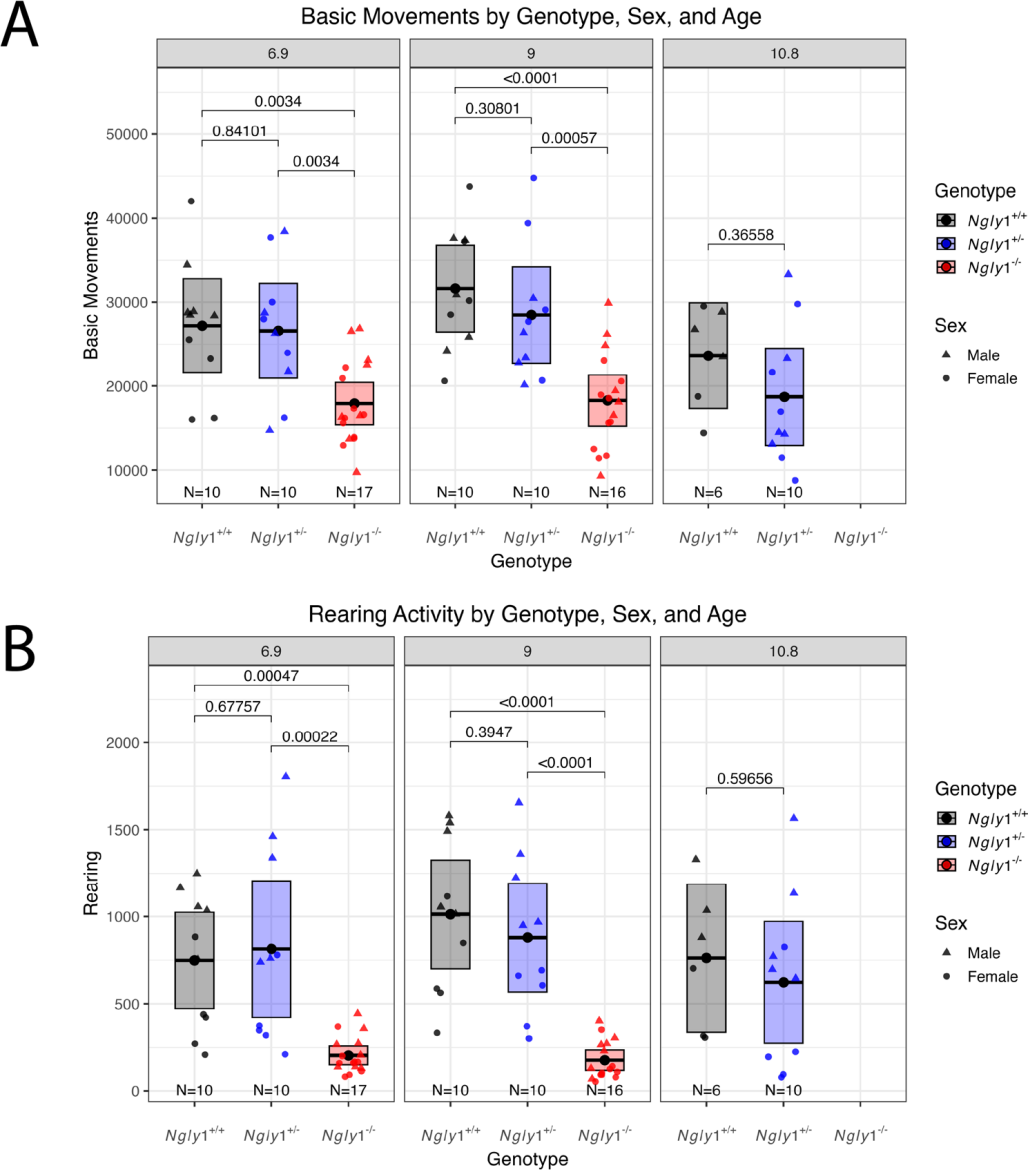
Locomotor open field test of *Ngly1*⁺^/^⁺, *Ngly1*⁺^/^⁻, and *Ngly1*⁻^/^⁻ rats Over Study Course. Locomotor testing at 6.9, 9, and 10.8 months of age for each of the 3 rat cohorts was performed by placing the animals into a Hamilton-Kinder enclosure and monitoring the number of basic movement and number of rearings for 15 min. *Ngly1*⁻^/^⁻ rats were not tested at 10.8 months of age due to premature mortality or deteriorating health in this cohort. The total basic movements (**A**) and the total number of rearing events (**B**) for each animal that completed the full 15-minutes sessions were totaled and plotted; the mean basic movements or rearing number per group is indicated as a dot-line within the box plot and the upper and lower edges represent 95% confidence interval (CI) (Mean ± CI). The p-value is labeled between genotypes at each time point and the number of animals (N) is indicated under each boxplot. Statistics: linear mixed model. M, male; F, female

Locomotor activity was assessed for all 3 rat cohorts at 6.9 and 9 months of age in an open field test using a Hamilton-Kinder enclosure, where horizontal (basic movement) and vertical (rearing) movements were recorded via infrared beam breaks over a 15-minute session. In the open field test, basic movement and number of rearings were both significantly lower in *Ngly1*⁻^/^⁻ rats compared to *Ngly1*⁺^/^⁺ and *Ngly1*⁺^/^⁻ rats at both 6.9-month and 9-month time points (Fig. 4). No significant difference in basic movements or number of rearings was observed between *Ngly1*⁺^/^⁺ and *Ngly1*⁺^/^⁻ rats at any of the tested time points (6.9, 9, and 10.8 months; Fig. 4).

At 6.9 months of age, *Ngly1*⁻^/^⁻ rats showed a markedly lower number of basic movements (17,909.7 ± 1,193.5, *N* = 17) compared to *Ngly1*⁺^/^⁺ (27,195.0 ± 2,463.6, *N* = 10, *p* = 0.0034, LMM) and *Ngly1*⁺^/^⁻ (26,579.2 ± 2,495.3, *N* = 10, *p* = 0.0034, LMM) rats. A similar pattern was observed in rearing activity, where *Ngly1*⁻^/^⁻ rats displayed a significantly lower number of rearings (204.4 ± 25.2, *N* = 17) than both *Ngly1*⁺^/^⁺ (748.6 ± 122.0, *N* = 10, *p* = 0.00047, LMM) and *Ngly1*⁺^/^⁻ (813.9 ± 173.3,*N* = 10, *p* = 0.00022, LMM) cohorts.

At 9.0 months of age, *Ngly1*⁻^/^⁻ rats continued to exhibit significantly reduced number of basic movements (18,266.8 ± 1,431.5, *N* = 16) compared to *Ngly1*⁺^/^⁺ (31,616.8 ± 2,285.0, *N* = 10, *p* < 0.0001, LMM) and *Ngly1*⁺^/^⁻ (28,474.9 ± 2,547.9, *N* = 10, *p* = 0.00057, LMM) rats. As was also observed for rats 6.9 months of age, *Ngly1*⁻^/^⁻ rats at 9.0 months of age displayed a significantly lower number of rearings (176.8 ± 27.8, *N* = 16) than either *Ngly1*⁺^/^⁺ (1,013.2 ± 138.0, *N* = 10, *p* < 0.0001, LMM) or *Ngly1*⁺^/^⁻ (879.1 ± 137.9, *N* = 10, *p* < 0.0001, LMM) rats.

Data from *Ngly1*⁻^/^⁻ rats were not available for a comparison of basic movement and number of rearings at 10.8 months due to the premature mortality of some animals and the significant deterioration of the surviving animals; however, *Ngly1*⁺^/^⁺ and *Ngly1*⁺^/^⁻ rats were tested at this time point. No significant differences in either basic movements or number of rearings were observed between *Ngly1*⁺^/^⁺ and *Ngly1*⁺^/^⁻ rats at any age tested, suggesting that loss of a single *NGLY1* allele does not impair gross locomotor behavior as assessed in the locomotor open field test. Both male and females *Ngly1*⁻^/^⁻ rats showed significantly lower basic movement and rearing measurements than their *Ngly1*⁺^/^⁺ and *Ngly1*⁺^/^⁻ counterparts at all time points. Interestingly, there was a significant difference in rearing between males and females for both the *Ngly1*⁺^/^⁺ and *Ngly1*⁺^/^⁻ genotypes but not between males and females of the *Ngly1*⁻^/^⁻ genotype.

In the Functional Observational Battery (FOB), a test of neurobehavioral behavior, *Ngly1*⁻^/^⁻ rats exhibited impairments in rearing (as assessed for 1-minute by a scorer), righting reflexes, and gait/mobility when compared to *Ngly1*⁺^/^⁺ and *Ngly1*⁺^/^⁻ rats at approximately 7 and 9 months of age (Supplemental Fig. 1). No significant differences were observed in other FOB parameters (data not shown). As with the locomotor open field test, there were no differences detected in the FOB between *Ngly1*⁺^/^⁺ and *Ngly1*⁺^/^⁻ rats at any time point (data not shown).

Based on cage side clinical observations, *Ngly1*⁻^/^⁻ rats displayed a consistent neurological phenotype at study initiation (~ 6 months of age) that was consistent with the previous characterization of this Ngly1 deficient rat model [13, 14]. Specifically, the *Ngly1*⁻^/^⁻ rats exhibited uncoordinated gait, hindlimb weakness (often asymmetric), bruxism, splayed limbs, head tilt, and labored breathing. Additional observations exclusive to *Ngly1*⁻^/^⁻ animals in this study included malocclusion, thin body condition, low head carriage, skin discoloration with scabbing, and dental discoloration. Overall, these phenotypes progressively worsened over time.

### *Ngly1*⁻^/^⁻ rats exhibit consistently elevated GNA levels in both plasma and CSF

**Fig. 5:**
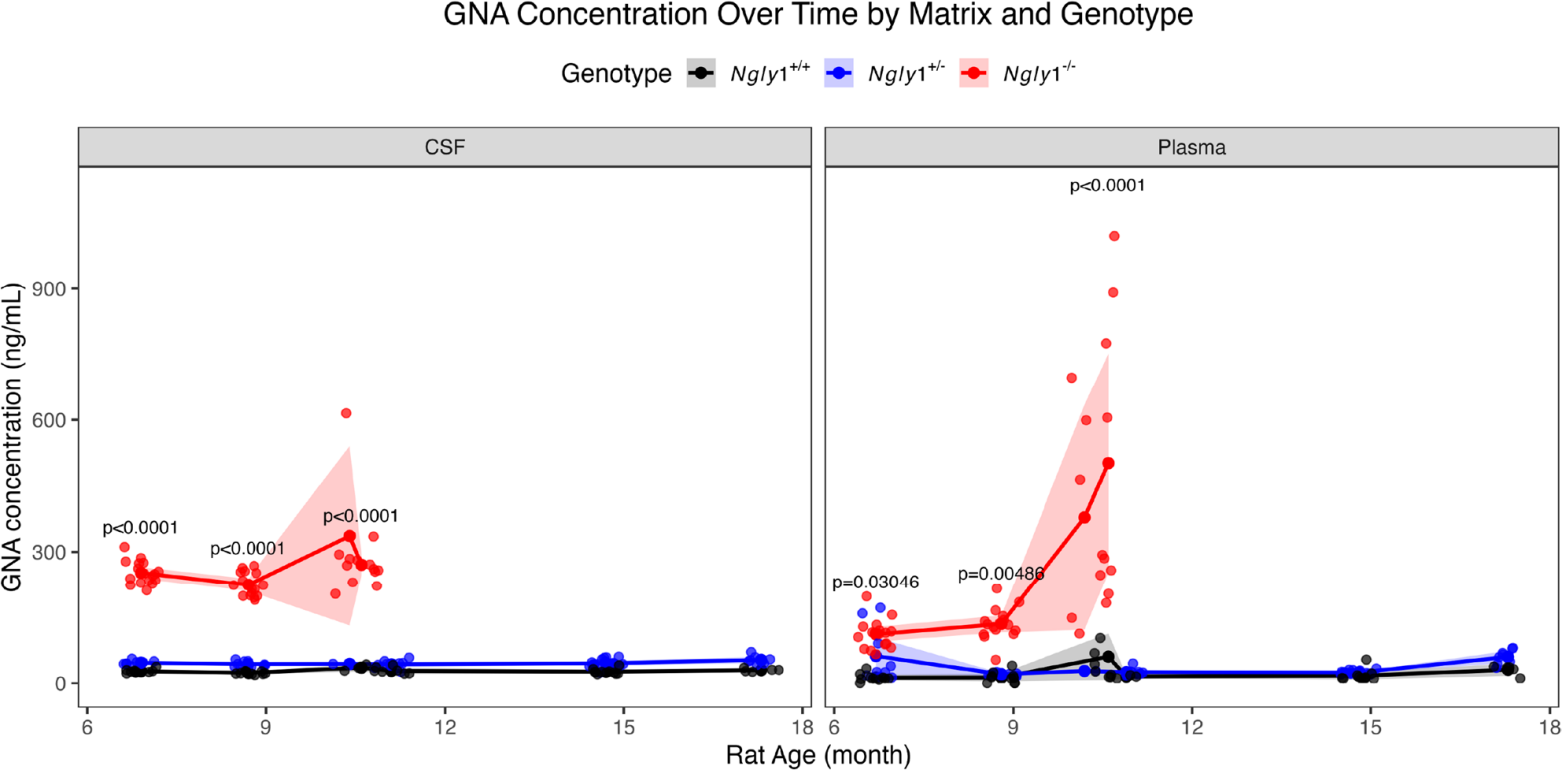
Longitudinal GNA levels of *Ngly1*⁺^/^⁺, *Ngly1*⁺^/^⁻, and *Ngly1*⁻^/^⁻ rats in CSF and Plasma over time. Longitudinal GNA biomarker levels were quantified in CSF and plasma from *Ngly1*⁺^/^⁺, *Ngly1*⁺^/^⁻, and *Ngly1*⁻^/^⁻ rats. CSF and plasma samples from *Ngly1*⁻^/^⁻ rats were available only at the first 3 timepoints (~ 6.5, ~ 9, and ~ 11 months), and plasma collected at the final time point (11 months) showed signs of visible hemolysis. Solid lines connect mean GNA concentrations for each genotype across time. Shaded areas represent 95% confidence intervals. Displayed p-values indicate *Ngly1*⁺^/^⁺ vs. *Ngly1*⁻^/^⁻ comparisons at each time point, derived from a linear mixed model. *Ngly1*⁺^/^⁺, N = 10; *Ngly1*⁺^/^⁻, N = 10; *Ngly1*⁻^/^⁻, N = 14 ~ 18 depending on survival to the timepoint indicated

Longitudinal analyses of GNA levels in both cerebrospinal fluid (CSF) and plasma were conducted in *Ngly1*⁻^/^⁻, *Ngly1*⁺^/^⁺, and *Ngly1*⁺^/^⁻ rats at the following time points: 6.2–7.1 months, 8.7–9.2 months, and 10.6–11.3 months (Fig. 5). Hemolysis was noted in the samples at the 10.6–11.3 month timepoint in the *Ngly1*⁻^/^⁻ rats which has the potential to artificially increase GNA concentration in a sample. Throughout the observation period, plasma GNA levels were consistently higher in *Ngly1*⁻^/^⁻ rats compared with either *Ngly1*⁺^/^⁺ or *Ngly1*⁺^/^⁻ rats at each time point. Interestingly, GNA levels in the plasma of *Ngly1*⁻^/^⁻ rats demonstrated an age-dependent increase from 6.5 months of age to their death or humane euthanasia at 12 months of age, while plasma GNA levels in *Ngly1*⁺^/^⁺ and *Ngly1*⁺^/^⁻ rats remained at similar, low levels throughout the study.

*Ngly1*⁻^/^⁻ rats also exhibited consistently elevated CSF GNA levels (252.2 ± 6.3 ng/mL, 224.5 ± 6.6 ng/mL, 336 ± 73 ng/mL) compared to *Ngly1*⁺^/^⁺ (27.5 ± 1.3 ng/mL, 23.6 ± 0.9 ng/mL, and 35.6 ± 2.9 ng/mL)and *Ngly1*⁺^/^⁻ (46.5 ± 1.5 ng/mL, 43.7 ± 1.8 ng/mL, 43.1 ± 2.7 ng/mL) rats across all 3 evaluated time points. In contrast to plasma GNA levels, CSF GNA levels remained stable within each cohort over time. Notably, *Ngly1*⁺^/^⁻ rats showed modestly higher CSF GNA levels than *Ngly1*⁺^/^⁺ controls at all time points.

### The neuroinflammatory markers IBA-1 and GFAP exhibit regional specific elevations in *Ngly1*⁻^/^⁻ rat brain

**Fig. 6:**
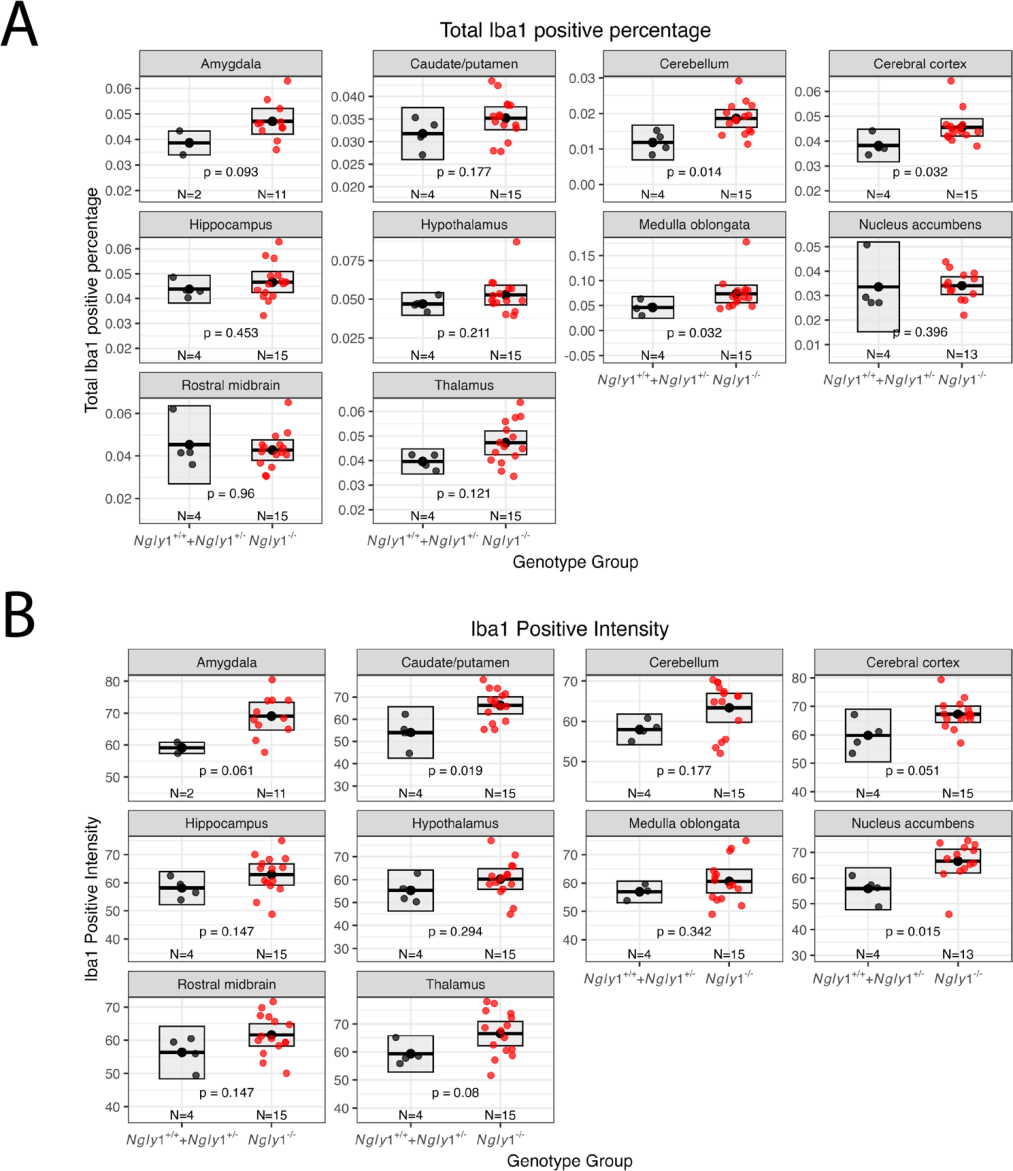
IBA-1 percentage and intensity in different brain regions. Tissue slices from rat brains were fixed, paraffin embedded, sliced, and immunostained with anti-IBA-1 antibodies. The slides were then scanned and analyzed using imaging software to quantify the number of IBA-1-positive cells and to calculate the percentage of cells with detectable IBA-1 expression (**A**) by normalizing to total cell count (DAPI). The intensity of each individual IBA-1 positive cells was averaged for each brain region (**B**). Each dot represents the average of 8 slices per brain of one animal. The mean per group is indicated as a dot-line within the box plot and the upper and lower edges represent 95% confidence interval (CI) (Mean ± CI). Statistical significance was calculated using unpaired two-tailed t-test

Brain tissues from *Ngly1*⁺^/^⁺ rats (*N* = 2), *Ngly1*⁺^/^⁻ rats (*N* = 2) that were terminated at 12 months of age, and *Ngly1*⁻^/^⁻ (*N* = 15) rats that died or were humanly euthanized at 12 months of age were analyzed for neuroinflammation using the astrocytic marker GFAP (Glial Fibrillary Acidic Protein) and the microglial marker IBA-1 (Fig. 6). Due to limited sample sizes for the *Ngly1*⁺^/^⁺ and *Ngly1*⁺^/^⁻ groups (*N* = 2 for each group) and a lack of statistically significant detectable phenotypic or physiological differences in any assessment between them, these two genotypes (4 rats in total) were combined into one group (*Ngly1*⁺^/^⁺ + *Ngly1*⁺^/^⁻) for comparison with the *Ngly1*⁻^/^⁻ rat brain tissues given minimal differences observed between *Ngly1*⁺^/^⁺ and *Ngly1*⁺^/^⁻ rats in other assessments. The analysis of the GFAP and IBA-1 staining included (1) the percentage of GFAP- or IBA-1–positive cells relative to the total number of cells (DAPI-positive cells) in each brain region, and (2) the average immunofluorescence intensity of GFAP or IBA-1 staining (Fig. 6).

Brain tissue from *Ngly1*⁻^/^⁻ rats exhibited a significantly higher positive percentage of IBA-1 positive cells than brain tissue from *Ngly1*⁺^/^⁺ and *Ngly1*⁺^/^⁻ rats in several brain regions: cerebellum (1.86 ± 0.12% vs. 1.18 ± 0.15%, *p* = 0.014, t-test), cerebral cortex: (4.56 ± 0.16% vs. 3.82 ± 0.21%, *p* = 0.032, t-test), and medulla oblongata (7.32 ± 0.83% vs. 4.60 ± 0.68%, *p* = 0.032, t-test) (Fig. 6A). *Ngly1*⁻^/^⁻ rats also exhibited a trend toward higher IBA-1-positive cell percentage (4.71 ± 0.23%) compared to *Ngly1*⁺^/^⁺ + *Ngly1*⁺^/^⁻ rats (3.87 ± 0.47%; *p* = 0.093) in the amygdala.

The IBA-1 staining intensity in *Ngly1*⁻^/^⁻ rat brain tissue was also significantly higher than in *Ngly1*⁺^/^⁺ and *Ngly1*⁺^/^⁻ rats in the following brain regions: caudate/putamen (66.28 ± 1.79 vs. 54.03 ± 3.64 units, *p* = 0.019, t-test), nucleus accumbens (66.59 ± 2.10 vs. 55.85 ± 2.56 units, *p* = 0.015, t-test), amygdala (69.08 ± 1.95 vs. 59.10 ± 1.74 units, *p* = 0.061, t-test), cerebral cortex (67.24 ± 1.32 vs. 59.74 ± 2.92 units, *p* = 0.051, t-test), and thalamus (66.55 ± 2.04 vs. 59.31 ± 2.04 units, *p* = 0.080) (Fig. 6B).

*Ngly1*⁻^/^⁻ rat brains tissue showed significantly higher positive percentage of GFAP positive cells than *Ngly1*⁺^/^⁺ and *Ngly1*⁺^/^⁻ rats in the amygdala (23.06 ± 0.61% vs. 11.66 ± 3.35%, *p* = 0.038, t-test), but this was not observed in other brain tissue regions (Supplemental Fig. 2).

These data indicate significant, region-specific neuroinflammation in *Ngly1*⁻^/^⁻ rat brain tissue, with clear statistical differences in certain areas and additional regions exhibiting trends. This suggests broad inflammatory disease pathology, potentially contributing to neuropathological outcomes observed in NGLY1 Deficiency.

### Hematological and histopathological analysis in *Ngly1*⁻^/^⁻ rat

A trend of increased large unstained cells (LUC) and monocytes was observed in *Ngly1*⁻^/^⁻ rat hematology compared to *Ngly1*⁺^/^⁺ and *Ngly1*⁺^/^⁻ rats (Supplemental Fig. 3). All other hematology measurements in *Ngly1*⁻^/^⁻ rats were similar to *Ngly1*⁺^/^⁺ and *Ngly1*⁺^/^⁻ rats. Histopathological analysis of central and peripheral nervous tissues of *Ngly1*⁻^/^⁻ rats (including the rats found dead prematurely) revealed several abnormalities not seen in *Ngly1*⁺^/^⁺ or *Ngly1*⁺^/^⁻ rats. These included CNS mineralization in certain brain regions, eosinophilic inclusions within the medulla oblongata, spinal cord gray matter, and dorsal root ganglia (DRG), signs of neurodegeneration in DRG, and axonal degeneration in spinal cord tracts, DRG, and sciatic nerve (data not shown). These results were consistent with previous observations from *Ngly1*⁻^/^⁻ rat brain tissue [13].

## Discussion

The assessment of aging in *Ngly1*⁻^/^⁻ rats in the reported study provides a comprehensive characterization of the disease over time that mirrors the progressive neurodegeneration observed in patients with NGLY1 Deficiency. Specifically, the pronounced motor deficits, premature mortality, and neuroinflammatory markers observed in these rats align with what is known about phenotypic progression and neurodegeneration in patients [19], underscoring the model’s translational relevance. In addition to the previously described early postnatal mortality observed prior to weaning at PND 21, we observed another major mortality event between 7 ~ 10 months of age in *Ngly1*⁻^/^⁻ rats that has not been previously reported [13, 14]. This data is consistent with reported deaths in patients during the course of an NGLY1 Natural History Study [7] and with the reported median lifespan of 14.6 years (unpublished data, Grace Science Foundation), underscoring the need for early intervention in patients with NGLY1 Deficiency.

The study’s finding that aging *Ngly1*⁻^/^⁻ rats continue to have elevated GNA levels in CSF and plasma further validates GNA as a robust and sustained diagnostic substrate derived biomarker for NGLY1 Deficiency [16]. However, the presence of hemolysis in the terminal *Ngly1*⁻^/^⁻ plasma samples may have confounded results in plasma in this study, artificially elevating GNA concentrations in that matrix at late timepoints. This elevation is likely due to the higher cytosolic GNA concentration that would be detectable only if cells are lysed and cytosol is present in the hemolysed plasma sample. As GNA is NGLY1 substrate derived, it will accumulate in the cytosol where NGLY1 typically functions. To discern whether this increase in elevation at end of life is real, plasma GNA concentrations will need to be further assessed in future studies. Studies of human GNA in NGLY1 Deficiency patients did not show increases in plasma GNA over time in prospective NGLY1 natural history studies [7, 16]. If GNA does show in future studies to be linked to age or end of life, this could be due to stress and cell lysis resulting in higher detectable GNA levels. The heterozygous animals in the current study showed a slight elevation of GNA compared to wild-type controls, similar to previous studies [16]. While not statistically significant, it suggests partial haploinsufficiency in carriers of mutant *NGLY1* alleles. As NGLY1 Deficiency carriers appear clinically normal, any biological significance of *NGLY1* haploinsufficiency may only occur in combination with other conditions [28] or may have as yet unidentified symptomatic associations. Genome-wide association studies or phenomics studies may help assess *NGLY1* as a modifier of clinical outcomes as additional pathogenic genetic variants for NGLY1 Deficiency are identified [9].

Phenotypic assessments in this study began at approximately 6 months of age. Based on the results, *Ngly1*⁻^/^⁻ rats appear to have already reached near-maximal motor impairment at this age. For example, rotarod latency was close to zero at 6.9 months (Fig. 3), consistent with prior reports of progressive decline [14]. Although rearing activity remained stable between 6.9 and 10.4 months, it was already significantly reduced compared to *Ngly1*⁺^/^⁺ and *Ngly1*⁺^/^⁻ littermates at that time point (Fig. 4). In contrast, the significant reduction in basic movements observed after 6 months was not present in younger animals, suggesting ongoing functional deterioration beyond early adulthood [14]. NGLY1 Deficiency patients show an increasing developmental gap compared to age-normative neurotypical peers either through deterioration or maintenance at a level of profound impairment in motor and cognitive domains [7]. The parallel deterioration reported in humans and rats supports the use of gross motor readouts in both species for the assessment of potential therapies. Improvement in any measure of motor function after therapeutic intervention in either species is likely to be indicative of a positive effect. However, this study does suggest that, due to the deterioration in the *Ngly1*⁻^/^⁻ rats leading to shortened lifespan, there is limited utility of gross motor readouts in the rat model of NGLY1 Deficiency beyond ~ 7 months of age.

In the current study, male *Ngly1*⁻^/^⁻ rats exhibited a marked reduction in body weight growth compared to *Ngly1*⁺^/^⁻ and *Ngly1*⁺^/^⁺ controls, whereas *Ngly1*⁻^/^⁻ female rats did not show significant differences from either their *Ngly1*⁺^/^⁻ or *Ngly1*⁺^/^⁺ counterparts. While compelling, there is limited human data to assess sex-related disparities. Studies of NGLY1 Deficient patients did not explicitly report sex differences in growth, likely due to small sample sizes [10, 11]. However, one recent natural history study showed male patients were more frequently underweight (over half of boys fell below the 3rd weight percentile, versus only ~ 29% of girls) [9]. Future studies in rats could assess the impact of sex hormones, metabolic regulation, the hormonal system, and developmental timing to understand sex-specific differences and overcome the limited patient pool available in humans, clarifying how sex-specific factors contribute to growth and weight trajectories in NGLY1 Deficiency [9, 13].

The neuroinflammation observed in *Ngly1*⁻^/^⁻ rats exhibiting premature mortality or clinical deterioration that required human euthanasia, characterized by increased expression of IBA-1 and GFAP in specific brain regions, suggests an inflammatory component to the disease’s pathology. This is consistent with previous reports of IBA-1 and ubiquitin pathology in younger *Ngly1*⁻^/^⁻ rats [13] and with postmortem findings from NGLY1 Deficiency patients showing neuronal inclusions and Purkinje cell loss [19]. The neuroinflammation in rat cortex and the observed DRG pathology is also consistent with human autopsy findings of eosinophilic cytoplasmic inclusions in the cortex and DRG [19]. In one human autopsy study [19], both eosinophilic inclusions and ubiquitin immuno-positive inclusions were found in the spinal cord, suggesting that the eosinophilic inclusions observed in the current rat study likely correspond to similar ubiquitin- or glycan-positive aggregates, although confirmatory staining would be needed to verify this directly. Interestingly, the neuroinflammation was mostly restricted to the thalamus in younger *Ngly1*⁻^/^⁻ rats [13] but seemed to progress with time and was found to be more widespread across multiple brain regions in the older rats observed in this study. The neuroinflammation and neurodegeneration suggest a potential causal link to the progressive motor and neurological decline observed in both *Ngly1*⁻^/^⁻ rats and human NGLY1 Deficiency patients. Neuroinflammation localized to specific CNS regions can impact motor function related to these regions. For example, pronounced microgliosis and astrogliosis in the cerebellum has been directly linked to cerebellar degeneration and ataxic motor deficits in chronic neuroinflammation models [29]. Microglial activation in the spinal cord, motor cortex, and medulla oblongata correlates with more severe motor deterioration and motor neuron clinical symptoms in amyotrophic lateral sclerosis (ALS) patients [30]. The elevated IBA-1 and GFAP expression observed in the cerebellum, cortex, and medulla of aging *Ngly1*⁻^/^⁻ rats may contribute to the further decline of motor function in older *Ngly1*⁻^/^⁻ rats. Given such inflammatory responses are also common in other neurodegenerative disorders there is the potential for shared pathogenic mechanisms that may be therapeutically targeted [20, 21].

In addition to the limits discussed above, the number of assessments and samples available for histological examination restricted the study. Additional functional cognitive tests (i.e. analysis of learning and memory), more quantitative functional motor assessments (e.g. gait), or additional neurophysiological examinations (Nerve Conduction Velocity, EEG, seizure assessment, sleep wake cycle) could provide additional insight into the progression of neurodegeneration in this animal model. In addition, the small sample size of the histology control cohort and limited tissue selection leaves some question as to the magnitude of the phenotypes observed in this study. Future studies should more quantitatively characterize the neurodegenerative phenotypes identified in this analysis.

In conclusion, the *Ngly1*⁻^/^⁻ rat model, when studied longitudinally through its lifespan, effectively recapitulates key aspects of human NGLY1 Deficiency, offering a valuable disease model that can be used to evaluate potential therapeutic interventions. The integration of molecular, biochemical, and histopathological analyses in this model enhances the understanding of the disease progression and highlights the need for therapeutic interventions.

## Materials and methods

### Animals

The Sprague-Dawley *Ngly1*⁻^/^⁻ rat was previously generated by deleting approximately 2.6 kb in exon 11 and exon 12 and a 3′ flanking region of the *Ngly1* gene via CRISPR-Cas9 gene editing technology [13]. Animal care procedures and experiments conformed to the United States Department of Agriculture (USDA) Animal Welfare Act (Code of Federal Regulations, Title 9 [9 CFR], Parts 1, 2, and 3) and are under the strict oversight of the Institutional Animal Care and Use Committee (IACUC), Ethics Committee (EC) and Animal Welfare Body (AWB) at Charles River Labs. Rats were pair-housed where possible. Animals were separated during designated procedures/activities and as required for monitoring and/or health purposes, as deemed appropriate by the principal investigator and/or clinical veterinarian. Animals were housed in solid-bottom cages with nonaromatic bedding. Fluorescent lighting was provided via an automatic timer for approximately 12 h per day. The basal diet was block Lab Diet Certified Rodent Diet #5002, PMI Nutrition International. Tap water was offered ad libitum to all animals via an automatic water system. Veterinary care was available throughout the course of the study, and animals were examined by the veterinary staff as warranted by clinical signs or other changes. Treatment of the animal(s) for minor injuries or ailments was approved when such treatment did not affect fulfillment of the study objectives. This study was conducted in compliance with the ARRIVE (Animal Research: Reporting of In Vivo Experiments) guidelines.

### Mortality/cageside observations, clinical observations, and humane euthanasia

Animals were observed within their cage twice daily for morbidity, mortality, injury, and availability of food and water. Any animals in poor health were identified for further monitoring and possible euthanasia. Poor health was determined by the observation that the animal was in overt pain/distress or appeared moribund and was beyond the point where recovery appeared reasonable. Animals in poor health were euthanized for humane reasons in accordance with the American Veterinary Medical Association (AVMA) Guidelines on Euthanasia.

Animals were removed from the cage for detailed clinical observation monthly. Observations will include evaluation of the skin, fur, eyes, ears, nose, oral cavity, thorax, abdomen, external genitalia, limbs and feet, respiratory and circulatory effects, autonomic effects such as salivation, nervous system effects including tremors, convulsions, reactivity to handling, and unusual behavior.

### Animal genotyping

A PCR genotyping assay that targeted the *Ngly1* gene locus was used to differentiate between *Ngly1*⁺^/^⁺, *Ngly1*⁺^/^⁻, and *Ngly1*⁻^/^⁻ rats. All rats were produced as previously described [16]. After genotype was confirmed, and equal number of animals per sex were selected randomly for inclusion in the study.

### Rotarod, locomotor, and FOB testing

Rotarod, locomotor, and FOB testing were conducted on animals without knowledge on the part of the testers of the treatment groups (blinded evaluations), and animals were randomized with respect to testing order to minimize bias and potential order effects.

The accelerating rotarod test is designed to measure balance, coordination, physical condition, and motor learning. Rats underwent rotarod testing using the San Diego Rotor-Rod Rod System. Conditioning sessions were performed on the morning of or a day prior to the actual testing and involved the animal being placed on the rotarod set to a constant speed of − 4 rpm (the minus sign indicates that the rod is moving in a direction that causes the rat to ambulate away from the observer) for 4 min. During the actual test, animals were placed on a rotating rod programmed to accelerate from 4 to 40 rpm at a rate of 0.15 revolutions per second over the course of 4 min. For animals staying on the rotarod for the entire 4-min period, a time of 240 s was recorded as the fall time. The elapsed time from the start of the trial to the animal’s fall was recorded digitally by the instrument. Each animal was evaluated in three trials with at least 15 min between trials. The average of all three trials is reported for each animal.

Locomotor testing was performed by placing the animals into a Hamilton-Kinder enclosure. The monitoring duration of basic movements and rearing activity was 15 min, with each activity measurement consisting of a count of the number of horizontal or vertical beam breaks respectively. General linear model with genotypes, time point, and route of administration as effects was used for statistical analysis comparing the averaged fall time (rotarod) and rearing activity for rotarod and locomotor, respectively.

FOB testing was conducted on all animals without knowledge on the part of the testers of the treatment status of each animal. Each animal was observed for a minimum of 3 min in a black Plexiglas open-field observation box. Parameters evaluated were based on those outlined by Moser and coworkers [22, 23].

### Pathological analysis

Brains and other tissues for histology were preserved in 10% neutral buffered formalin (NBF) for 24–48 h and transferred to 70% ethanol for up to an additional 72 h prior to processing to paraffin. Slices (2 mm) were generated from prepared tissue blocks and mounted on slides for histopathological evaluation or immunohistochemistry staining by a pathologist. Evaluation was based on the pathologist scoring from 0 (no findings, normal) to 5 (severe). No images were taken or other quantification carried out.

### Quantitative GNA measurements

Rat terminal CSF was collected by intracisternal magna (ICM) puncture, drawn with a syringe, frozen, and later analyzed. Plasma samples were isolated from blood taken via the sublingual vein (in-life collections) or cardiac puncture (terminal collection). Hemolysis was noted if present. Tissue samples were taken upon sacrifice without perfusion. Samples were frozen, dissected to 20- to 50-mg pieces while frozen, homogenized, and analyzed. Prepared rat tissue samples were analyzed as previously described [15]. Briefly, samples were homogenized in phosphate buffered saline (PBS), mixed with internal standard and acetonitrile, and centrifuged. An aliquot of each supernatant was separated via high-pressure liquid chromatography (HPLC) (Shimadzu VP Series 10 System, Shimadzu Corporation, Japan) and analyzed via tandem mass spectrometry (MS/MS) (Applied Biosystems/MDS SciEx API 4000, Danaher Corporation, Washington DC). Detection and accuracy were assessed in surrogate matrices (PBS + bovine serum albumin (BSA), charcoal stripped serum). Each surrogate matrix was spiked with 30 or 300 ng/mL of GNA, processed, and analyzed to determine recovery and accuracy.

### Statistical analysis

Analyses were performed using R (Version 3.6.3 for Mac, R Core Team) or GraphPad Prism version 10.3.1 for Mac (GraphPad Software, www.graphpad.com*).* Data were analyzed using a combination of parametric and non-parametric methods, selected based on distribution and experimental design. Normality was assessed visually and, where applicable, using the Shapiro-Wilk test. No a priori power calculations were performed due to the exploratory nature of this aging study and the lack of existing data on late-onset phenotypes in Ngly1⁻^/^⁻ rats. Cohort sizes were chosen to balance biological replication with expected attrition due to premature mortality, and statistical models were used to maximize analytical power by accounting for repeated measures, sex, and inter-animal variability.

For repeated-measures data such as body weight, motor functions, and GNA levels across time points, linear mixed models (LMMs) were used with genotype, time point, and sex as fixed effects, and animal ID as a random effect. These models were implemented using the lme4 package, with post hoc pairwise comparisons.

Immunohistochemistry data were analyzed using unpaired two-tailed t-tests.

Statistical significance was set at *p* < 0.05 was considered significant and is either directly labeled on the figure or designated with an asterisk in all figures: ns, *p* > 0.05; *p ≤ 0.05; **p ≤ 0.01; ***p ≤ 0.001; ****p ≤ 0.0001. Specific statistical tests are noted in each figure legend. Data are presented as mean ± SEM unless otherwise stated.

All behavioral, biomarker, and immunohistochemistry analyses were conducted with animals of both sexes, and sex was included as a fixed factor in the statistical models. Body weight was analyzed separately by sex due to known differences in body weight between sexes.

## Supplementary Information

Below is the link to the electronic supplementary material.

Supplementary Material 1

Supplementary Material 2

Supplementary Material 3

Supplementary Material 4

Supplementary Material 5

Supplementary Material 6

## Data Availability

Data is provided in the Supplementary Data file and analysis has been described in the methods section of the manuscript.

## References

[1] Tadashi Suzuki H, Fujihira. NGLY1: A fascinating, multifunctional molecule, Biochimica et Biophysica Acta (BBA) - General Subjects, Volume 1868, Issue 2, 2024, 130379, ISSN 0304–4165. 10.1016/j.bbagen.2023.130379 37951368

[2] Manole A, Wong T, Rhee A, Novak S, Chin SM, Tsimring K, Paucar A, Williams A, Newmeyer TF, Schafer ST, Rosh I, Kaushik S, Hoffman R, Chen S, Wang G, Snyder M, Cuervo AM, Andrade L, Manor U, Lee K, Jones JR, Stern S, Marchetto MC, Gage FH. NGLY1 mutations cause protein aggregation in human neurons. Cell Rep. 2023;42(12):113466. 10.1016/j.celrep.2023.113466. Epub 2023 Nov 30. PMID: 38039131; PMCID: PMC10826878. 38039131 10.1016/j.celrep.2023.113466 PMC10826878

[3] William F, Mueller P, Jakob HS, Ghidelli-Disse SC-MS, Ordonez D, Boesche M, Bantscheff M, Collier P, Haase B, Benes V, Paulsen M, Sehr P, Lewis J, Drewes G, Lars M, Steinmetz. Loss of N-Glycanase 1 Alters Transcriptional and Translational Regulation in K562 Cell Lines, G3 Genes|Genomes|Genetics, Volume 10, Issue 5, 1 May 2020, Pages 1585–1597. 10.1534/g3.119.401031 PMC7202010 32265286

[4] Huang C, Harada Y, Hosomi A, Masahara-Negishi Y, Seino J, Fujihira H, Funakoshi Y, Suzuki T, Dohmae N, Suzuki T. Endo-β-N-acetylglucosaminidase forms N-GlcNAc protein aggregates during ER-associated degradation in Ngly1-defective cells. Proc Natl Acad Sci U S A. 2015;112(5):1398–403. 10.1073/pnas.1414593112. Epub 2015 Jan 20. PMID: 25605922; PMCID: PMC4321286. 25605922 10.1073/pnas.1414593112 PMC4321286

[5] Tomlin FM, Gerling-Driessen UIM, Liu YC, Flynn RA, Vangala JR, Lentz CS, Clauder-Muenster S, Jakob P, Mueller WF, Ordoñez-Rueda D, Paulsen M, Matsui N, Foley D, Rafalko A, Suzuki T, Bogyo M, Steinmetz LM, Radhakrishnan SK, Bertozzi CR. Inhibition of NGLY1 inactivates the transcription factor Nrf1 and potentiates proteasome inhibitor cytotoxicity. ACS Cent Sci. 2017;3(11):1143–55. 10.1021/acscentsci.7b00224. Epub 2017 Oct 25. PMID: 29202016; PMCID: PMC5704294. 29202016 10.1021/acscentsci.7b00224 PMC5704294

[6] Levy RJ, Frater CH, Gallentine WB, Phillips JM, Ruzhnikov MR. Delineating the epilepsy phenotype of NGLY1 deficiency. J Inherit Metab Dis. 2022;45(3):571–583. 10.1002/jimd.12494. Epub 2022 Mar 11. PMID: 35243670. 10.1002/jimd.12494 35243670

[7] Tong S, Ventola P, Frater CH, Klotz J, Phillips JM, Muppidi S, Dwight SS, Mueller WF, Beahm BJ, Wilsey M, Lee KJ. NGLY1 deficiency: a prospective natural history study. Hum Mol Genet. 2023;32(18):2787–96. 10.1093/hmg/ddad106. PMID: 37379343; PMCID: PMC10481101. 37379343 10.1093/hmg/ddad106 PMC10481101

[8] Panneman DM, Wortmann SB, Haaxma CA, van Hasselt PM, Wolf NI, Hendriks Y, Küsters B, van Emst-de Vries S, van de Westerlo E, Koopman WJH, Wintjes L, van den Brandt F, de Vries M, Lefeber DJ, Smeitink JAM, Rodenburg RJ. Variants in NGLY1 lead to intellectual disability, myoclonus epilepsy, sensorimotor axonal polyneuropathy and mitochondrial dysfunction. Clin Genet. 2020;97(4):556–66. 10.1111/cge.13706. Epub 2020 Jan 30. PMID: 31957011; PMCID: PMC7078978. 31957011 10.1111/cge.13706 PMC7078978

[9] Stanclift CR, Dwight SS, Lee K, Eijkenboom QL, Wilsey M, Wilsey K, Kobayashi ES, Tong S, Bainbridge MN. NGLY1 deficiency: estimated incidence, clinical features, and genotypic spectrum from the NGLY1 registry. Orphanet J Rare Dis. 2022;17(1):440. 10.1186/s13023-022-02592-3. PMID: 36528660; PMCID: PMC9759919. 36528660 10.1186/s13023-022-02592-3 PMC9759919

[10] Lam C, Ferreira C, Krasnewich D, Toro C, Latham L, Zein WM, Lehky T, Brewer C, Baker EH, Thurm A, Farmer CA, Rosenzweig SD, Lyons JJ, Schreiber JM, Gropman A, Lingala S, Ghany MG, Solomon B, Macnamara E, Davids M, Stratakis CA, Kimonis V, Gahl WA, Wolfe L. Prospective phenotyping of NGLY1-CDDG, the first congenital disorder of deglycosylation. Genet Med. 2017;19(2):160–8. 10.1038/gim.2016.75. Epub 2016 Jul 7. PMID: 27388694; PMCID: PMC7477955. 27388694 10.1038/gim.2016.75 PMC7477955

[11] Enns GM, Shashi V, Bainbridge M, Gambello MJ, Zahir FR, Bast T, Crimian R, Schoch K, Platt J, Cox R, Bernstein JA, Scavina M, Walter RS, Bibb A, Jones M, Hegde M, Graham BH, Need AC, Oviedo A, Schaaf CP, Boyle S, Butte AJ, Chen R, Chen R, Clark MJ, Haraksingh R, FORGE Canada Consortium, Cowan TM, He P, Langlois S, Zoghbi HY, Snyder M, Gibbs RA, Freeze HH, Goldstein DB. Mutations in NGLY1 cause an inherited disorder of the endoplasmic reticulum-associated degradation pathway. Genet Med. 2014;16(10):751-8. Epub 2014 Mar 20. Erratum in: Genet Med. 2014;16(7):568. Chen, Rui [added]. PMID: 24651605; PMCID: PMC4243708. 10.1038/gim.2014.22 10.1038/gim.2014.22 PMC4243708 24651605

[12] Stuut T, Popescu O, Oviedo A. N-Glycanase 1 deficiency is a rare cause of pediatric neurodegeneration with neuronal inclusions and liver steatosis. Cureus. 2021;13(10):e19126. 10.7759/cureus.19126. PMID: 34858763; PMCID: PMC8614178. 34858763 10.7759/cureus.19126 PMC8614178

[13] Yang K, Torres-Ramirez G, Dobbs N, Han J, Asahina M, Fujinawa R, Song K, Liu Y, Lin W, Oviedo A, Chen C, Zhu L, Mueller WF, Lee K, Suzuki T, Yan N. The STING pathway drives noninflammatory neurodegeneration in NGLY1 deficiency. J Exp Med. 2025;222(10):e20242296. 10.1084/jem.20242296. Epub 2025 Jul 11. PMID: 40644312; PMCID: PMC12249164. 40644312 10.1084/jem.20242296 PMC12249164

[14] Fujihira H, Masahara-Negishi Y, Tamura M, Huang C, Harada Y, Wakana S, Takakura D, Kawasaki N, Taniguchi N, Kondoh G, Yamashita T, Funakoshi Y, Suzuki T. Lethality of mice bearing a knockout of the Ngly1-gene is partially rescued by the additional deletion of the Engase gene. PLoS Genet. 2017;13(4):e1006696. 10.1371/journal.pgen.1006696. PMID: 28426790; PMCID: PMC5398483. 28426790 10.1371/journal.pgen.1006696 PMC5398483

[15] Asahina M, Fujinawa R, Nakamura S, Yokoyama K, Tozawa R, Suzuki T. Ngly1 ⁻^/^⁻ rats develop neurodegenerative phenotypes and pathological abnormalities in their peripheral and central nervous systems. Hum Mol Genet. 2020;29(10):1635–47. 10.1093/hmg/ddaa059. PMID: 32259258; PMCID: PMC7322575. 32259258 10.1093/hmg/ddaa059 PMC7322575

[16] Zhu L, Tan B, Dwight SS, Beahm B, Wilsey M, Crawford BE, Schweighardt B, Cook JW, Wechsler T, Mueller WF. AAV9-NGLY1 gene replacement therapy improves phenotypic and biomarker endpoints in a rat model of NGLY1 deficiency. Mol Ther Methods Clin Dev. 2022;27:259–71. PMID: 36320418; PMCID: PMC9593239. 36320418 10.1016/j.omtm.2022.09.015 PMC9593239

[17] Asahina M, Fujinawa R, Fujihira H, Masahara-Negishi Y, Andou T, Tozawa R, Suzuki T. JF1/B6F1 Ngly1⁻^/^⁻ mouse as an isogenic animal model of NGLY1 deficiency. Proc Jpn Acad Ser B Phys Biol Sci. 2021;97(2):89–102. 10.2183/pjab.97.005. PMID: 33563880; PMCID: PMC7897899. 33563880 10.2183/pjab.97.005 PMC7897899

[18] Mueller WF, Zhu L, Tan B, Dwight S, Beahm B, Wilsey M, Wechsler T, Mak J, Cowan T, Pritchett J, Taylor E, Crawford BE. GlcNAc-Asn is a biomarker for NGLY1 deficiency. J Biochem. 2022;171(2):177–186. 10.1093/jb/mvab111. Erratum in: J Biochem. 2022;171(4):469. doi: 10.1093/jb/mvac016. PMID: 34697629; PMCID: PMC8863169. 10.1093/jb/mvab111 PMC8863169 34697629

[19] Crawley JN. Behavioral phenotyping of rodents. Comp Med. 2003;53:140–6. 12784847

[20] Curzon P. 2009. The Behavioral Assessment of Sensorimotor.

[21] Amor S, Peferoen LA, Vogel DY, Breur M, van der Valk P, Baker D, van Noort JM. Inflammation in neurodegenerative diseases–an update. Immunology. 2014;142(2):151–66. 10.1111/imm.12233. PMID: 24329535; PMCID: PMC4008224. 24329535 10.1111/imm.12233 PMC4008224

[22] Glass CK, Saijo K, Winner B, Marchetto MC, Gage FH. Mechanisms underlying inflammation in neurodegeneration. Cell. 2010;140(6):918–34. 10.1016/j.cell.2010.02.016. PMID: 20303880; PMCID: PMC2873093. 20303880 10.1016/j.cell.2010.02.016 PMC2873093

[23] Moser VC, McCormick JP, Creason JP, MacPhail RC. Comparison of chlordimeform and carbaryl using a functional observational battery. Fundam Appl Toxicol. 1988;11(2):189–206. 10.1016/0272-0590(88)90144-3. PMID: 3146518. 10.1016/0272-0590(88)90144-3 3146518

[24] Moser VC. Rat strain- and gender-related differences in neurobehavioral screening: acute trimethyltin neurotoxicity. J Toxicol Environ Health. 1996;47(6):567 – 86. PMID: 8614024. 10.1080/009841096161546 10.1080/009841096161546 8614024

[25] Yoshida Y, Asahina M, Murakami A, Kawawaki J, Yoshida M, Fujinawa R, Iwai K, Tozawa R, Matsuda N, Tanaka K, Suzuki T. Loss of peptide:N-glycanase causes proteasome dysfunction mediated by a sugar-recognizing ubiquitin ligase. Proc Natl Acad Sci U S A. 2021;118(27):e2102902118. 10.1073/pnas.2102902118. PMID: 34215698; PMCID: PMC8271764. 34215698 10.1073/pnas.2102902118 PMC8271764

[26] Asahina M, Fujinawa R, Hirayama H, Yukitake H, Suzuki T. AAV9-mediated NGLY1 gene replacement suppresses non-epileptic convulsions in Ngly1⁻^/^⁻ rats. Biochem Biophys Res Commun. 2025;790:152823. 10.1016/j.bbrc.2025.152823. Epub 2025 Oct 24. PMID: 41176936. 41176936 10.1016/j.bbrc.2025.152823

[27] Hernandez AR, Truckenbrod LM, Campos KT, Williams SA, Burke SN. Sex differences in age-related impairments vary across cognitive and physical assessments in rats. Behav Neurosci. 2020;134(2):69–81. 10.1037/bne0000352. Epub 2019 Dec 30. PMID: 31886694; PMCID: PMC7078049. 31886694 10.1037/bne0000352 PMC7078049

[28] Reina-Llompart N, Serrano-López S, Torres-Iglesias G, Álvarez-Troncoso J. Tofacitinib Improves Motor Symptoms in Parkinsonism Associated with a Heterozygous NGLY1 Variant and Autoimmune Disease. Eur J Case Rep Intern Med. 2025;12(11):005886. PMID: 41229635; PMCID: PMC12604884. 10.12890/2025_005886 10.12890/2025_005886 PMC12604884 41229635

[29] Gyengesi E, Rangel A, Ullah F, Liang H, Niedermayer G, Asgarov R, Venigalla M, Gunawardena D, Karl T, Münch G. Chronic microglial activation in the GFAP-IL6 mouse contributes to Age-Dependent cerebellar volume loss and impairment in motor function. Front Neurosci. 2019;13:303. 10.3389/fnins.2019.00303. PMID: 31001075; PMCID: PMC6456818. 31001075 10.3389/fnins.2019.00303 PMC6456818

[30] Brettschneider J, Toledo JB, Van Deerlin VM, Elman L, McCluskey L, Lee VM, Trojanowski JQ. Microglial activation correlates with disease progression and upper motor neuron clinical symptoms in amyotrophic lateral sclerosis. PLoS ONE. 2012;7(6):e39216. 10.1371/journal.pone.0039216. Epub 2012 Jun 14. PMID: 22720079; PMCID: PMC3375234. 22720079 10.1371/journal.pone.0039216 PMC3375234

