# Supplementary material for: Progressive neurodegeneration, motor decline, and premature mortality in aging Ngly1 deficient rats
Source: Orphanet J Rare Dis. 2026 Feb 20;21:110. doi: 10.1186/s13023-026-04261-1 (PMC13032507; doi:10.1186/s13023-026-04261-1)
Supplement: Supplementary file 6 — Supplementary Material 6 [file 13023_2026_4261_MOESM6_ESM.docx]

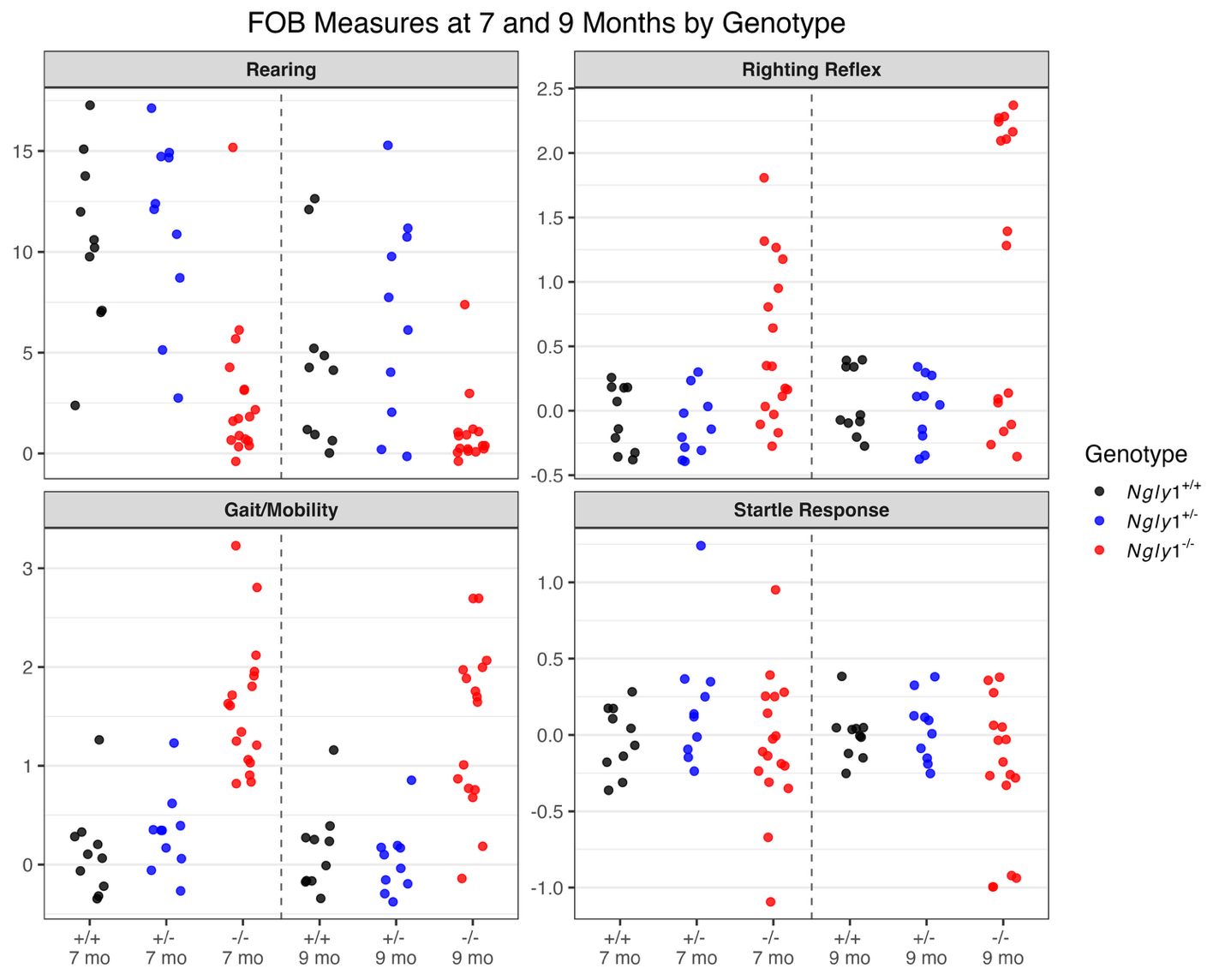


**Supplemental Figure 1. Selected FOB outcomes**

FOB measures — Rearing, Righting Reflex, Gait/Mobility, and Startle Response — were assessed in rats of three genotypes (*Ngly1*^+/+^, ^+/−^, and ^−/−^) at 7 and 9 months of age. Y-axis values for Rearing represents the number of events observed by an experimenter within 1 minute. For Gait/Mobility and Startle Response, values represent severity scores, with 0 indicating normal and 5 indicating severely impaired function.


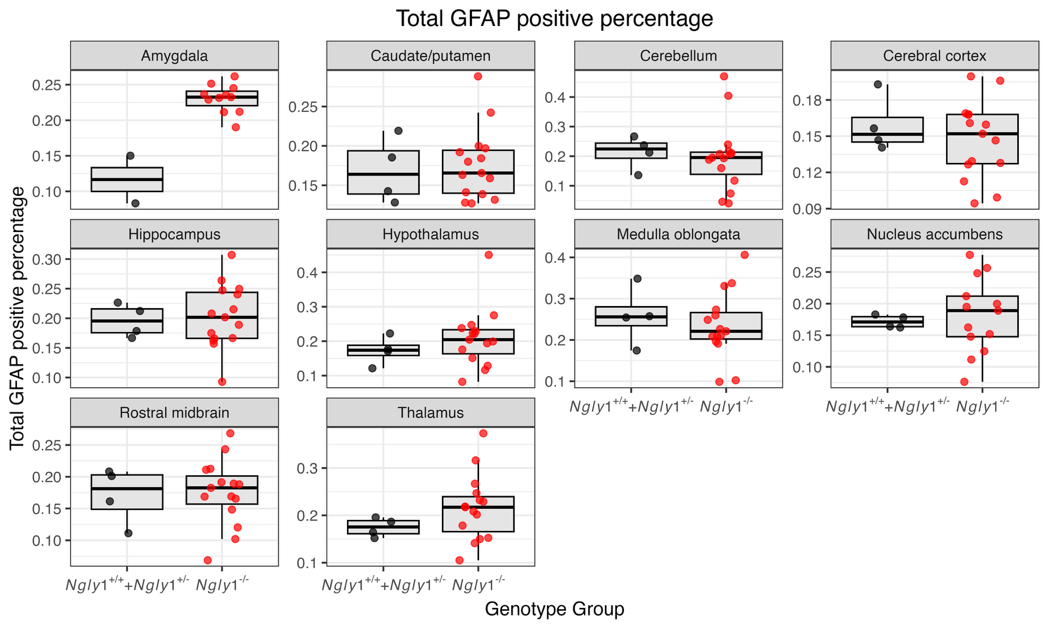


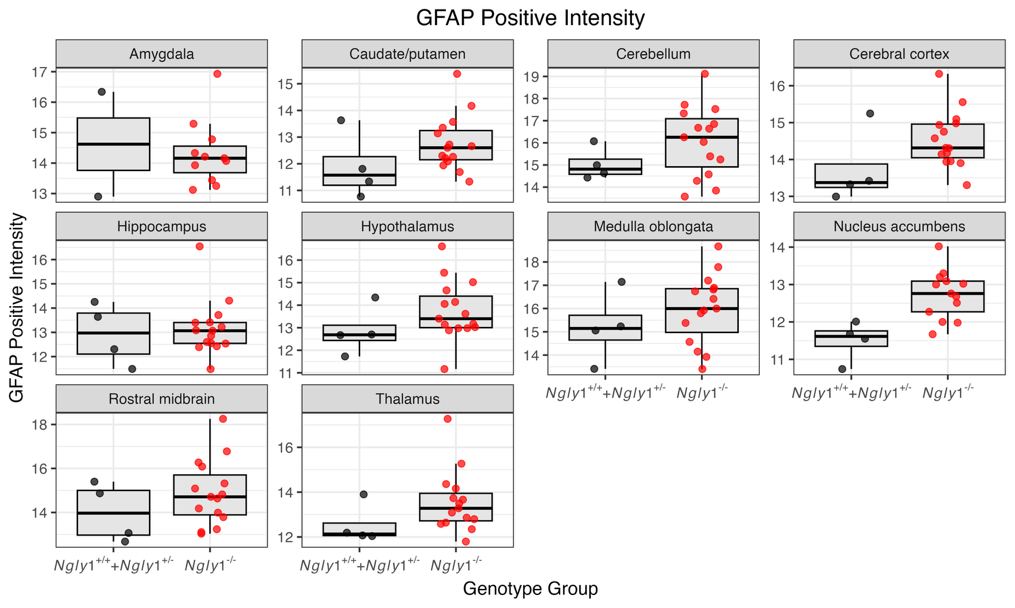


**Supplemental Figure 2. GFAP percentage and intensity**

Tissue slices from rat brains were fixed, paraffin embedded, sliced, and immunostained with anti-GFAP antibodies. The slides were then scanned and analyzed using imaging software to quantify the number of GFAP-positive cells and to calculate the percentage of cells with detectable GFAP expression by normalizing to total cell count (DAPI). Each dot represents the average of 8 slices per brain. Data are presented as mean box plots, where the box represents the interquartile range (25th–75th percentile), the line indicates the mean, whiskers show the minimum and maximum values, and outliers (if any) are plotted individually. Statistical significance was calculated using unpaired two-tailed t-test.

Hematology By Genotype


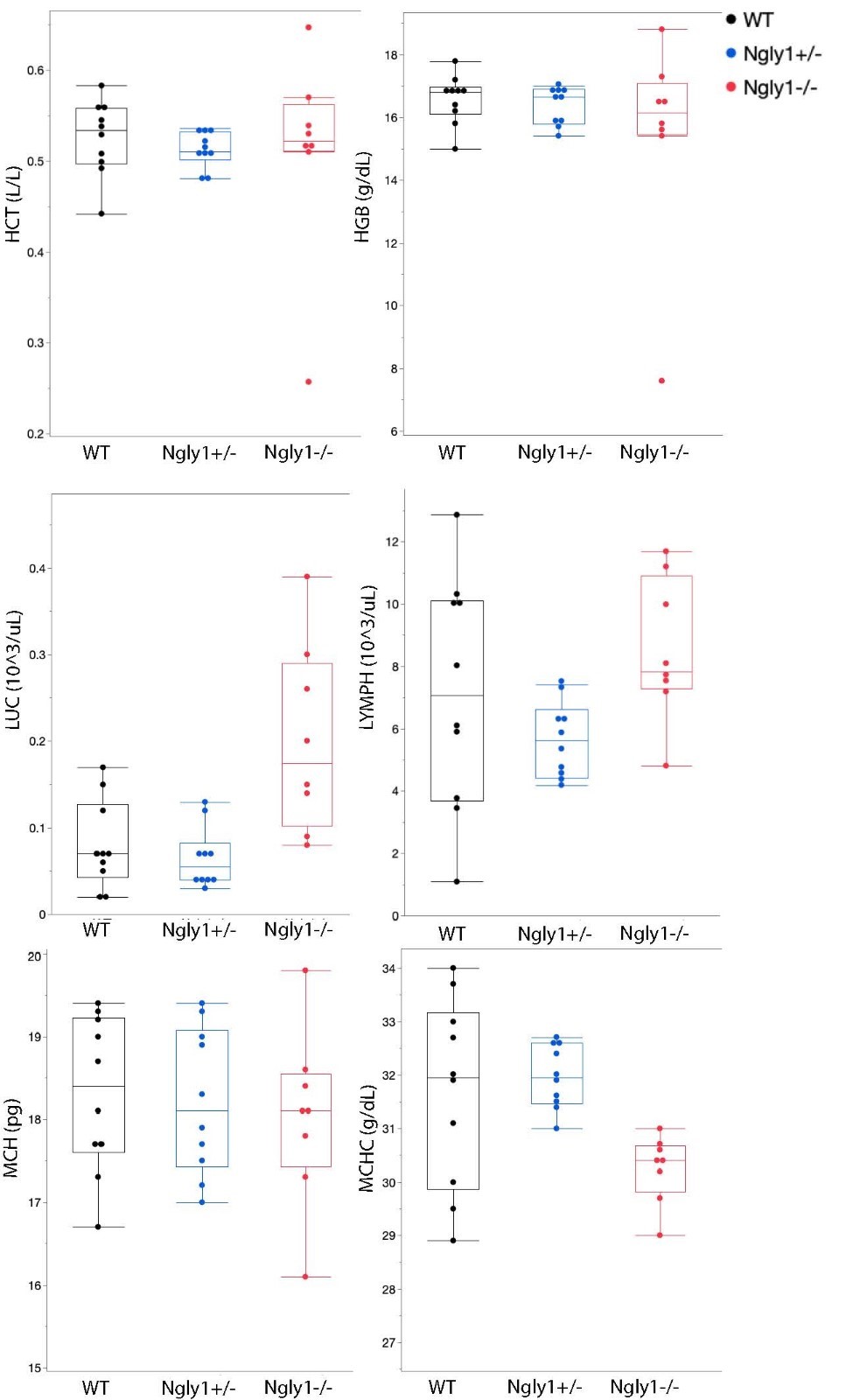


**Supplemental Figure 3. Hematology analysis**

Hematology was performed on samples obtained at two time points for *Ngly1*^+/+^and *Ngly1*^+/-^ rats as indicated in the graph and for *Ngly1*^-/-^ rats at 10-11 months of Age. HCT, hematocrit; HGB, hemoglobin; MCH, mean corpuscular hemoglobin; LUC, large unstained cells; LYMPH, lymphocyte; MCHC, mean corpuscular hemoglobin concentration.
